# A Miniature Modular Fluorescence Flow Cytometry System

**DOI:** 10.3390/bios14080395

**Published:** 2024-08-16

**Authors:** Shaoqi Huang, Jiale Li, Li Wei, Lulu Zheng, Zheng Shi, Shiwei Guo, Bo Dai, Dawei Zhang, Songlin Zhuang

**Affiliations:** 1 Engineering Research Center of Optical Instrument and System, the Ministry of Education, Shanghai Key Laboratory of Modern Optical System, University of Shanghai for Science and Technology, Shanghai 200093, China; 211180048@st.usst.edu.cn (S.H.); 223330706@st.usst.edu.cn (J.L.); weilioptic@usst.edu.cn (L.W.); llzheng@usst.edu.cn (L.Z.); slzhuang@usst.edu.cn (S.Z.); 2 Department of Gastrointestinal Surgery, The First Affiliated Hospital of Naval Medical University, Shanghai 200433, China; mdshizheng@outlook.com; 3 Department of Hepatobiliary Pancreatic Surgery, The First Affiliated Hospital of Naval Medical University, Shanghai 200433, China; gestwa@163.com

**Keywords:** modular microfluidics, flow cytometry, fluorescence detection, drug screening

## Abstract

Fluorescence flow cytometry is a powerful instrument to distinguish cells or particles labelled with high-specificity fluorophores. However, traditional flow cytometry is complex, bulky, and inconvenient for users to adjust fluorescence channels. In this paper, we present a modular fluorescence flow cytometry (M-FCM) system in which fluorescence channels can be flexibly arranged. Modules for particle focusing and fluorescence detection were developed. After hydrodynamical focusing, the cells were measured in the detection modules, which were integrated with in situ illumination and fluorescence detection. The signal-to-noise ratio of the detection reached to 33.2 dB. The crosstalk among the fluorescence channels was eliminated. The M-FCM system was applied to evaluate cell viability in drug screening, agreeing well with the commercial cytometry. The modular cytometry presents several outstanding features: flexibility in setting fluorescence channels, cost efficiency, compact construction, ease of operation, and the potential to upgrade for multifunctional measurements. The modular cytometry provides a multifunctional platform for various biophysical measurements, e.g., electrical impedance and refractive-index detection. The proposed work paves an innovative avenue for the multivariate analysis of cellular characteristics.

## 1. Introduction

Flow cytometry (FCM) is an important instrumental tool that allows the multi-parametric analysis of single cells in a flowing stream. The development of flow cytometry is of great benefit to the fields of biological research and disease diagnosis [1,2,3]. Fluorescence detection is an essential approach in the FCM to investigate cells with high-specificity labeling. The cellular status in terms of, for example, viability, apoptosis and cell cycle, can be evaluated, and the expression levels of biomarkers can be quantitively assessed [4,5]. The fluorescence detection system in conventional FCM has a complex, bulky layout, in which emission light at different wavelengths is separated by sets of dichroic mirrors and optical filters. Since the fluorescence channels are arranged in spectral order, it is not sufficiently flexible to update optical filtering systems.

In addition, a variety of forms of advanced cytometry have been demonstrated. Measuring the refractive indices of cells is a straightforward way to understand intracellular mass and concentration, providing an important insight for biological models [6,7]. Angle-resolved scattered light detection was adopted in cytometry, fulfilling the demands of on-chip applications [8,9]. Electrical impedance cytometry was presented to probe unlabeled cells for dielectric properties [10,11,12]. Multiple biophysical metrics, including membrane physiology and cytoplasmic conductivity, can be revealed by impedance cytometry [13,14]. Moreover, deformability cytometry harnesses hydrodynamic forces or applies external forces on cells to induce measurable deformation, based on which the mechanical properties of cells, such as surface friction, viscosity and elasticity, can be thoroughly studied [15,16,17,18]. The abovementioned demonstrations have achieved great success in the microfluidic fields [19,20,21,22]. Microfluidic cytometry has the potential to be modularized and integrated with other forms for multi-parametric analysis.

Modular microfluidics have attracted significant research interest due to their flexibility, reconfigurability and functionality in use [23,24,25,26,27,28]. A group of modules with standard structures is assembled to form a system for a particular application. In addition to the structural modules, functional modules, e.g., the valve, photodetector and camera, are of great significance for fluid control and signal acquisition [29,30]. Modular microfluidics have been applied to immunoassays, organoid research and chemical synthesis [31,32,33]. Nevertheless, constructing a reconfigurable cytometry using modular microfluidics remains a challenge, which might be attributed to the complexity of the system with the current layout and the lack of proper modules.

Herein, we present a modular flow cytometry (M-FCM) system for high-throughput cell detection. Modules for cell focusing and fluorescence detection are proposed. The layout of the fluorescence detection and signal acquisition was changed to a parallel style. It is very flexible, making it possible to arrange and update the detection modules for different combinations of fluorescence channels. The detection modules exhibit a high signal-to-noise ratio. The optical crosstalk among the fluorescence channels is efficiently eliminated. Furthermore, the M-FCM is employed in the demonstration of drug screening, evaluating the viability and the drug resistance of cells. The M-FCM is designed with high flexibility in arranging and updating fluorescence channels, satisfying customized experimental requirements. The M-FCM system is quite cost-efficient compared to commercial FCM, while ensuring good performance. The system has the potential to be upgraded for other types of biophysical detection, e.g., electrical impedance and refractive-index detection, making it useful for multivariate biomedical applications. We anticipate the integration of a diverse range of biophysical detection modules in M-FCM for biomedical research and clinical diagnosis.

## 2. Materials and Methods

### 2.1. Design and Fabrication of the M-FCM

**Figure 1:**
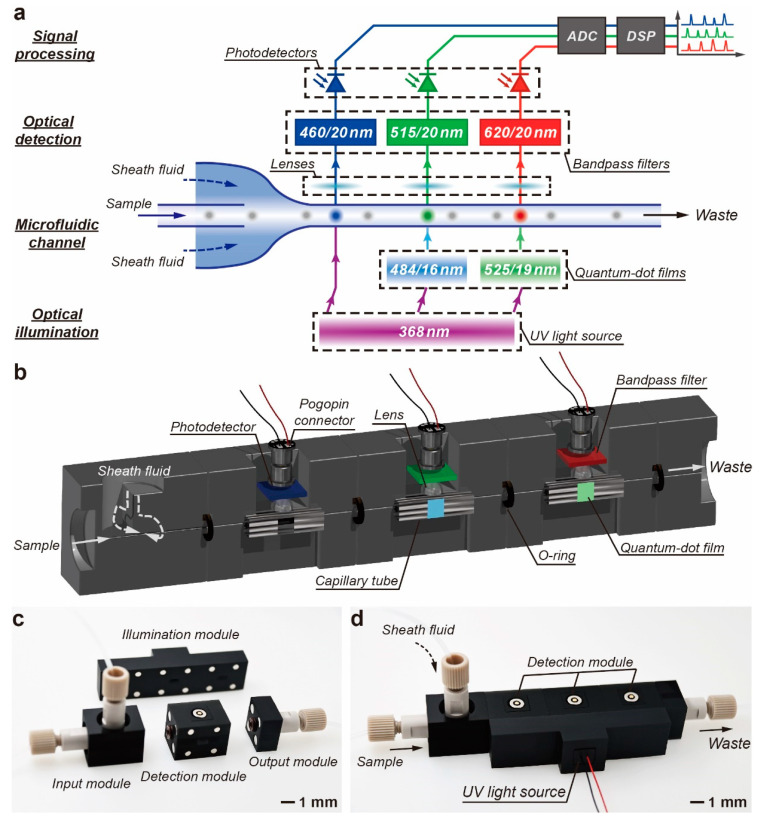
Concept of the M-FCM. (**a**) Schematic diagram of the M-FCM. (**b**) Cross-section illustration of the M-FCM. (**c**) Photograph of the key modules used in the M-FCM. (**d**) Photograph of the fully assembled M-FCM.

Figure 1a shows the schematic diagram of the M-FCM, which consists of four parts, including optical illumination, fluorescence detection, microfluidic channel and signal processing. A 368 nm UV LED (6565LG, Shenzhen Yuanming Technology Co., Ltd., Shenzhen, China) was employed as the light source to light up the samples and excite the quantum dot (QD) films to generate 484 nm and 525 nm light for illumination. The UV LED has an optical power of 280 mW. The sample is injected into the microchannel with two sheath flows. The 3D hydrodynamic focusing of the particles in the microchannel is calculated by COMSOL Multiphysics^®^ software (COMSOL, Inc., Burlington, MA, USA). Ten-micrometer--diameter particles were released from the input. Transport of diluted-species field was employed to characterize the distribution of sample and sheath flow in the microchannel. The particles are focused on the center of the cross section of the microchannel and pass through the detection window separately (Figure S1). Above each detection window, there is a lens and an optical filter to collect and filter out fluorescent emission. A photodetector (LSSPD-SMDB1.5, Beijing Lightsensing Technology Co., Ltd., Beijing, China) is followed to convert fluorescent signals to electrical signals. The photodetection is conducted in parallel for multiple fluorescence channels. After signal amplification and analog-to-digital conversion, the waveforms of the signals are plotted.

The structures of the main channel and key components are illustrated in Figure 1b. The entire M-FCM can be divided into several modules, including input module, detection modules, output module and illumination module, as shown in Figure 1c. The fully assembled M-FCM with three fluorescence channels has a footprint of 15 mm × 85 mm (Figure 1d). The modules were designed using computer-aided design software, Autodesk^®^ Inventor^®^ (Autodesk, Inc., San Rafael, CA, USA). The frames of the modules were fabricated with black biocompatible resin (BIO, BMF Precision Technology Co., Shenzhen, China) by a projection micro-stereolithography 3D printer (nanoArch^®^ P140, BMF Precision Technology Co., Shenzhen, China). The modules can be precisely aligned and tightly connected using neodymium iron boron magnets. O-ring was inserted in between the modules to avoid leakage of the flow. Polyetheretherketone adapters were used in the input and output modules to connect the external syringe pump and waste container via the tubes.

The illumination module is equipped with a UV LED and a 1 × 3 optical splitter, as shown in Figure S2. The module was 3D-printed. Liquid-state RTV silicone with 1:1 weight ratio of elastomer and curing agent (Gelest, Inc., Morrisville, NC, USA) was filled into the internal channels of the module and cured at 55 °C for 4 h, forming the optical waveguides. A 368 nm UV LED was installed at the input port. The output ports were aligned to the illumination area in the detection modules. The power from the optical splitter at each output port is approximately 60 mW, as illustrated in Figure S3.

The detection modules are highly integrated with a capillary tube, a hemispherical lens, an optical bandpass filter, a photodiode and a pogo-pin connector. The capillary tube was deposited with 300-nanometer-thick Ag film by magnetron sputtering deposition except for illumination and detection windows on the adjacent sides. The two windows have areas of 1.8 mm × 3 mm. The illumination window was coated with a QD film (LAB18, Kurt J. Lesker Co., Pittsburgh, PA, USA). The film was formed by dispersing QDs (QUVINK, Xiamen Bohr Technology Co., Ltd., Xiamen, China) in the UV-curable photoresist, spin-coating the QD solvent onto the capillary tube at 3500 rpm for 1 min and curing under UV exposure for 3 min. The capillary tube was sealed in the 3D-printed frame with polydimethylsiloxane (PDMS) with a 10:1 weight ratio between silicone prepolymer and cross linker. The central wavelength/bandwidths of the optical bandpass filters were 460/20 nm, 515/20 nm and 620/20 nm, respectively. All the filters had optical density of 6. The photodiodes were directly integrated on the pogo-pin connectors. The effective detection area is 2.25 mm^2^ and the responsivity is 0.43 A∙W^−1^. Each detection module measures 15 mm × 15 mm × 20 mm.

### 2.2. Characterization of Illumination Source

The UV LED was measured by a LED opto-electronic analyzer (Ever fine, ATA-500, Hangzhou, China). The peak wavelength and the full width at half maximum bandwidth of the UV LED are 368 nm and 15 nm, respectively (Figure S4a). The emission spectra of QD films were measured by a fluorescence spectrophotometer (FLS1000, Edinburgh Instruments Ltd., Livingston, UK) with a photomultiplier tube (R928, Hamamatsu Photonics, Shizuoka, Japan). The emission peaks of the QD films (Xiamen Bohr Technology Co., Ltd., Xiamen, China) are at 484 nm and 525 nm (Figure S4b,c) and the bandwidths are 16 nm and 19 nm, respectively.

### 2.3. Data Acquisition and Processing

After optical-to-electrical conversion, the signals were amplified to a range of 0 V to 10 V. Next, the analog-to-digital conversions were conducted at sampling rate of 1 MS s^−1^ and with 16-bit resolution using commercial data acquisition hardware (NI 9223 BNC, National Instruments, Austin, TX, USA). The data were calculated on a computer using a customized LabVIEW program (National Instruments, Austin, TX, USA). Denoising and averaging were conducted before peak detection and counting.

### 2.4. Cell Culture and Preparation

K562 and K562/ADR human myelogenous leukemia cell lines (Zhejiang Meisen Cell Technology Co., Ltd., Shaoxing, China) were cultured in Roswell Park Memorial Institute (RPMI) 1640 culture medium supplemented with 10% fetal bovine serum (FBS), 100·U·mL^−1^ penicillin and 100 μg mL^−1^ streptomycin. The drug resistance of K562/ADR cell line was maintained by introducing 500 ng mL^−1^ doxorubicin to the culture medium. All cells were incubated at 37 °C with 5% CO_2_.

Samples used for drug-screening experiment were prepared by treating the K562 and K562/ADR cells with 500 ng mL^−1^ doxorubicin for 6, 12 and 24 h, respectively. The samples were co-stained with 4′,6-diamidino-2-phenylindole (DAPI) (Thermo Fisher Scientific, Waltham, MA, USA), calcein acetoxymethyl ester (Calcein-AM) and propidium iodide (PI) (Dojindo Laboratories) for 20 min at 37 °C. The samples were divided into two groups. One group was examined by a commercial FCM (CytoFlex S, Beckman Coulter Inc, Pasadena, CA, USA). The examination results were processed using FlowJo_v10.8.1 software (Tree Star Inc., Ashland, OR, USA). Cell population gating was set based on FSC-A and SSC-A threshold to eliminate noncellular population and cellular debris. Clustered cells were removed from the samples by utilizing singlet cell gating based on FSC-A and FSC-H threshold. The other group was measured by M-FCM. The results were analyzed by customized LabVIEW and MATLAB (Matrix Laboratory, MathWorks Inc., Natick, MA, USA) programs.

## 3. Results and Discussion

### 3.1. Enhancement of the Fluorescence Detection

**Figure 2:**
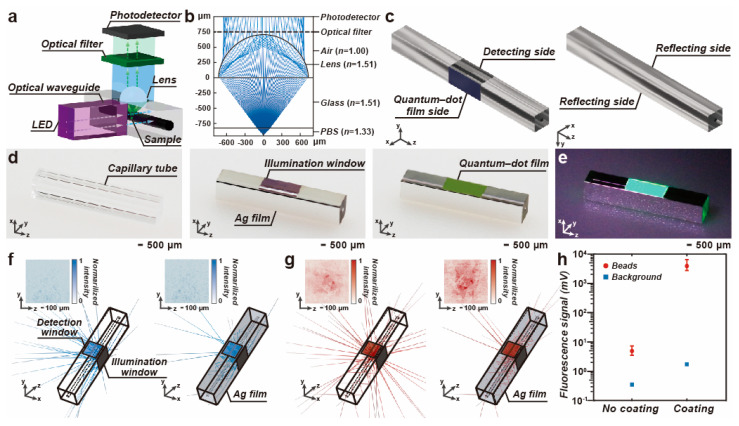
Calculation of the fluorescence detection in the compact detection module. (**a**) Configuration of the optical system in the detection module. (**b**) Simulation of the ray trajectories in the detection part of the optical system. (**c**) Schematic diagram of the capillary tube deposited with the Ag film and the QD film. (**d**) Fabrication procedure of the capillary tube. (**e**) The capillary tube with the Ag and QD films exposed in the UV light. (**f**) Intensity distribution projected on the photodetector and the ray tracing of the light emitted from the QD film when the capillary tube is deposited with and without the Ag film. (**g**) Ray tracing and the intensity distribution on the photodetector of the emission light from the fluorescent beads flowing through the microchannel when the capillary tube is without and with the Ag film. (**h**) The influence of the Ag film coating over the fluorescence detection. The measurement was repeated three times.

The optical setup of the fluorescence detection has a compact structure, as depicted in Figure 2a. The UV light is guided to the detection module via the optical waveguide and illuminates the QD film on the capillary tube. The QDs are lit up to emit a narrow-bandwidth light that is then used to excite the samples flowing through the capillary tube. A short focal-length hemispherical lens above the capillary tube collects the fluorescence. It is worth noting that precise focusing is required for the efficient collection of the fluorescence and high collimation of the output to the optical filter, whose extinction ratio of signal-to-background noise is sensitive to the incident angle. Figure 2b illustrates the result of the ray-tracing simulation. When the hemispherical lens attached to the capillary tube has the matched refractive index, the light from the lens passes through the optical filter perpendicularly, ensuring a high extinction ratio.

In addition, the capillary tube was deposited with the Ag film to enhance the fluorescence detection, as shown in Figure 2c. The fabrication procedure of the film deposition is shown in Figure 2d. The capillary tube was deposited with the 300-nanometer Ag film, except the illumination and detection windows. Next, the illumination window was coated with the QD films. Two types of QD are used for different-color illumination. The excitation light for the QDs is identical. Under 368 nm of UV illumination, the QD films emit blue and green light, respectively. Figure 2e demonstrates the bright green light emission from the QD film coated on the capillary tube.

The emission of the QD film is used to excite the samples labeled with the fluorophore. The fluorescence intensity is proportional to the intensity of the excitation. A high photoluminescence quantum yield from the QD films is preferred, which leads to high fluorescence emission from the samples. In addition, efficient use of the excitation light might also improve the fluorescence detection. Figure 2f illustrates the light distribution of the emission of the QD film and a point source that acts as a fluorescent bead centered at the microchannel under the detection window. When the capillary tube is covered with the Ag film, the emission of the QD film is trapped inside the capillary tube and multi-reflection occurs. The aggregation of the light contributes to a strong fluorescence excitation. Thus, the emission intensity of the fluorophore can be enhanced.

In addition, the omnidirectional fluorescence emission can be efficiently coupled to the detection window due to the internal reflection. The amount of fluorescence detected from the capillary tube deposited with the Ag film is larger than that from the tube without film deposition, as shown in Figure 2g. Meanwhile, the output of the QD emission is also increased. The degradation of the background noise cannot be neglected. To improve the signal-to-noise ratio, an operational amplifier-based high-pass filter is adopted in the signal processing for fluorescence signal amplification and low-frequency noise suppression.

Figure 2h depicts the measurement of the noise and the fluorescence output in the capillary tube with and without Ag film deposition. The noise was measured by exposing a 484-nanometer QD film under UV light, while the fluorescence signal was read out when chemiluminescent beads passed through the detection window and no excitation light was used, i.e., no background noise was present in the measurement. The output of the signal evidently increased. By comparison, the signal-to-noise ratio is improved by 21.8 dB if the capillary tube is deposited with the Ag film.

### 3.2. Evaluation of the Optical Crosstalk

In the conventional FCM, the samples labeled with multiple fluorophores were lit up by broadband light. Next, the fluorescence emission was separated into different optical paths for multi-channel detection by dichroic mirrors and optical filters arranged in series [34,35,36]. The optical system is bulky and complex. Moreover, the fluorescence intensity drops after the stage-by-stage fluorescence channel separation. In this work, the proposed scheme employs quartz capillary tubes, which are coated with QD and Ag films for in situ fluorescence excitation and detection. Multiple QD-based narrowband sources are used as the excitation for different fluorescence channels. The illumination and the detection are arranged in parallel.

**Figure 3:**
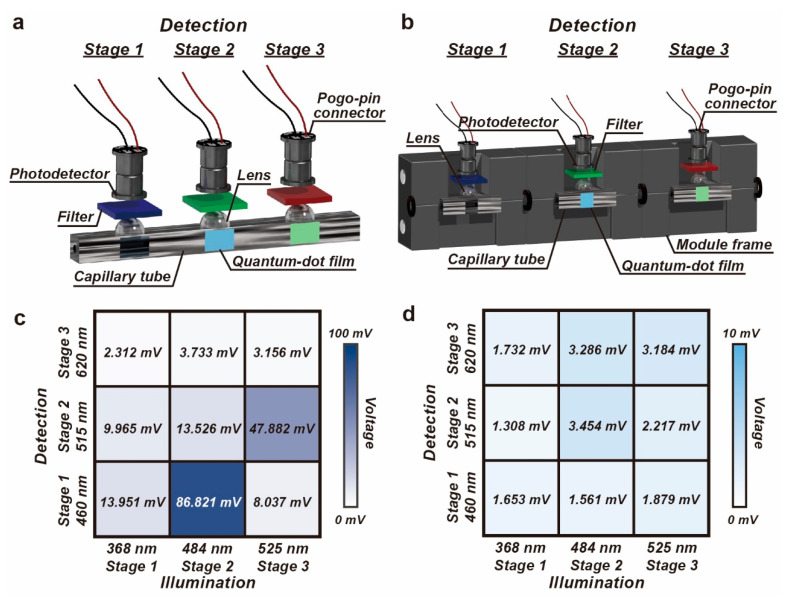
Evaluation of the optical crosstalk among the fluorescence channels. (**a**) Schematic diagram of the detection system using a single capillary tube. (**b**) Schematic diagram of the cascaded detection modules in the M-FCM. (**c**) Severe optical crosstalk occurs among the three fluorescence channels if a single capillary tube is used. (**d**) The optical crosstalk of the fluorescence detection in the M-FCM is negligible.

If the multiple fluorescence channels are integrated in a single capillary tube, as illustrated in Figure 3a, the excitation and emission light propagates along the tube. As shown in Figure 2e, the capillary tube functions as an optical waveguide and the end of the tube presents the green light that is generated from the QD film. The excitation light propagating along the tube is detected in the other fluorescence channels by mistake, resulting in the inaccurate measurement of the fluorescence emission. Hence, the propagation of the excitation and emission light should be forbidden, eliminating the optical crosstalk.

In the proposed scheme, a frame for the detection module is designed to fulfill the requirement of the integration of the capillary tube with the fluorescence detection components and the isolation of the light. The frames are 3D-printed in black, offering high absorbance over UV to visible bands (Figure S5). Short segments of the capillary tubes are embedded in the 3D-printed frames, and the propagation of the light can be efficiently obstructed, as shown in Figure 3b.

The optical crosstalk among the fluorescence channels is evaluated by measuring the output of the detection when the excitation light is lit up stage by stage. A UV LED was directly attached to the illumination window at each stage. During the illumination, the optical power was read out simultaneously from the three cascaded fluorescence channels. After the data acquisition, the LED was moved to the next stage for the illumination. When a single capillary tube is used for the three fluorescence channels, severe optical crosstalk occurs among the fluorescence channels, whose excitation and emission bands have overlaps in the spectrum. The optical crosstalk among the fluorescence channels using a single capillary tube and M-FCM was measured ten times, as listed in Supplementary Tables S1–S4. Figure 3c,d, Figures S6 and S7 show the background noise caused by the optical crosstalk. The noise suppression in the M-FCM improves by 25 times in comparison to that in the single tube. If the detection system is divided into a group of detection modules, the detection noise is very low, which is mainly attributable to the dark-current noise generated by the photodetector. Thus, the detection is not affected by the excitation from the other fluorescence channels and the crosstalk among the fluorescence channels is negligible. Furthermore, Supplementary Tables S2–S4 and Figure S7 demonstrate the optical crosstalk when the detection modules were arranged in different orders. Thanks to the isolation of the modular strategy, there is no influence from the arrangement over the optical performance.

### 3.3. Integration of the Detection Module

**Figure 4:**
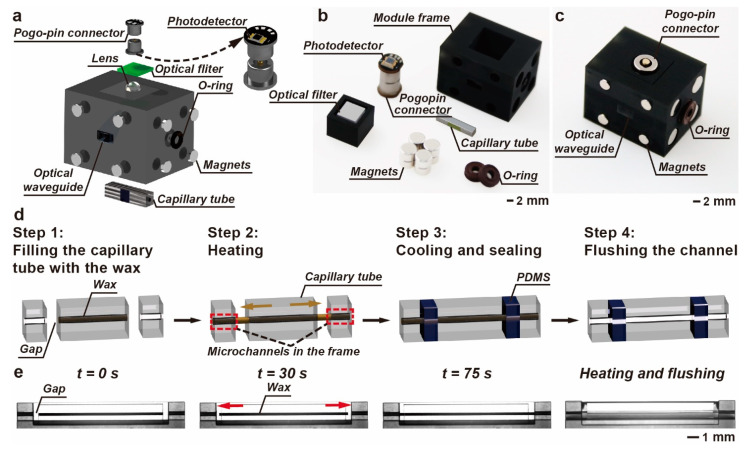
Design and fabrication of the detection module. (**a**) Exploded view of the detection module. (**b**) Photograph of the components used for the detection module. (**c**) The highly integrated detection module. (**d**) Embedding and sealing capillary tube in the detection module. (**e**) The montage of the thermal expansion of the wax and the spontaneous connection of the microchannels.

The detection module is integrated with microfluidic system, illumination system and detection system, as shown in Figure 4a–c. The frame was fabricated by projection micro-stereolithography 3D printing. The printing resolution is 10 μm, which is sufficiently high to precisely install the components and align the modules. There is a slot in front of the illumination window. The slot is filled with the transparent RTV silicone, which is used as an optical waveguide connecting the QD film and the output of the illumination module. The UV light can be efficiently guided to the QD film for the generation of the excitation light. The QD films for the excitation generation and the optical filters for the emission filtering can be customized as required. The optical components are fixed in the frame using black silicone.

The response of the fluorescence detection is determined by the minimum bandwidth, *BW_Min_*, of the photodetector, the amplifier and the data acquisition hardware. The duration of the detection can be approximately counted as the time that each sample passes by the window. Thus, the maximum flow rate can be estimated as follows:

(1) $$Q_{Max}=\frac{4\lambda fBW_{Min}S}{0.7\pi D}$$

where *λ* is the wavelength of the emission light, *f* is the focal length of the lens, *D* is the effective aperture of the lens and *S* is the cross-section area of the microchannel.

The microchannel in the detection module is not a single monolithic structure. The existence of gaps between the capillary tube and the frame leads to leakage. The gaps should be filled up, while the microchannel should remain unobstructed. Figure 4d illustrates the integration of the capillary tube into the frame. Before the installation, the capillary tube is fully filled with thermo-sensitive wax. The melting point of the wax is 55 °C and thermal expansion coefficient is 15%. Next, the capillary tube is placed into the frame, aligning the microchannel. The frame containing the capillary tube is heated up to 65 °C in the oven. The wax turns to liquid, with gradually increased volume. The wax slowly flows out of the capillary tube at the two ends and then crosses the gap without dispersing due to the high viscosity. Figure 4e and Video S1 demonstrate the expansion of the wax and the interconnection of the microchannels in the capillary tube and the frame. The capillary action drives the wax to enter the microchannels in the frame. When the wax is through the microchannel between the tube and the frame, the gaps are sealed with the PDMS. Finally, the wax is flushed away with sodium bicarbonate solution and dilute water. The microchannels in the capillary tube and the frame join up into a single line, ensuring that fluid flows freely throughout the detection module. No leakage is observed when the flow rate is up to 1 mL min^−1^.

### 3.4. Drug Screening Using the M-FCM

The M-FCM assembled with three detection modules was applied for the drug screening. The sheath fluid (Beckman B51503 CytoFLEX, Beckman Coulter Inc., USA) was first injected into the M-FCM to fill the microchannel. After stabilization, 10-microliter cell suspension with a concentration of 5000 cells μL^−1^ was injected into the M-FCM. Six groups of K562 and K562/ADR cells cultured with doxorubicin for 6, 12 and 24 h were used. The cells were labelled with three fluorescent tracers, i.e., DAPI, Calcein-AM and PI. The purpose of the DAPI was to count the total number of cells and the purpose of the Calcein-AM and the PI was to evaluate the viability and death of the cells, respectively. The flow rates of the sheath fluid and the cell suspension were 400 μL min^−1^ and 200 μL min^−1^, respectively. The fluid channel was cleaned using proteolytic enzyme solution (Coulter DxH Cleaner, Beckman Coulter Inc, USA) after use to avoid contamination.

**Figure 5:**
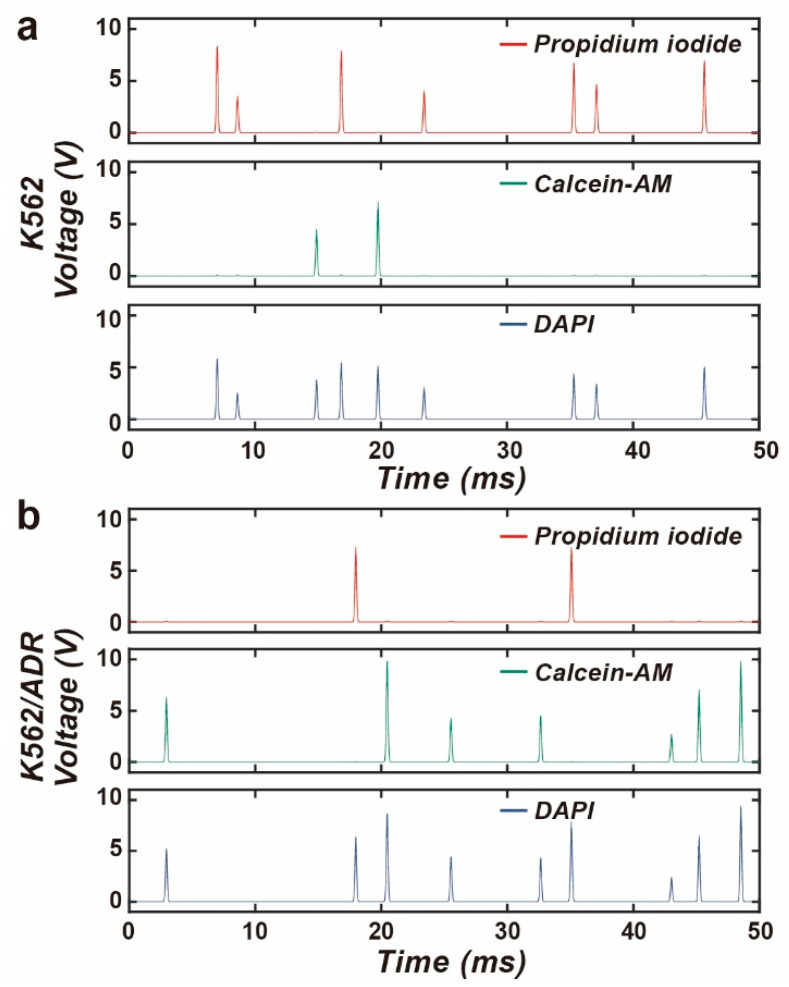
The waveforms for the three fluorescence channels detected by the M-FCM for (**a**) K562 cells and (**b**) K562/ADR cells after 24-h culture in the doxorubicin.

The three fluorescence channels operated in parallel, and the data were read out simultaneously. Figure 5 and Supplementary Figure S8 show the waveforms detected by the M-FCM for the K562 cells and K562/ADR cells cultured in the doxorubicin after 24 h. The background noise resulted from fluorescence excitation, optical crosstalk, photodetection and electrical amplification is suppressed well. The pulses representing the appearance of the fluorescence were clearly distinguished. The pulse width was about 1.5 ms. A large number of pulses were detected in the PI fluorescence channel for the K562 cells, while only a few were observed in the measurement for the K562/ADR cells.

**Figure 6:**
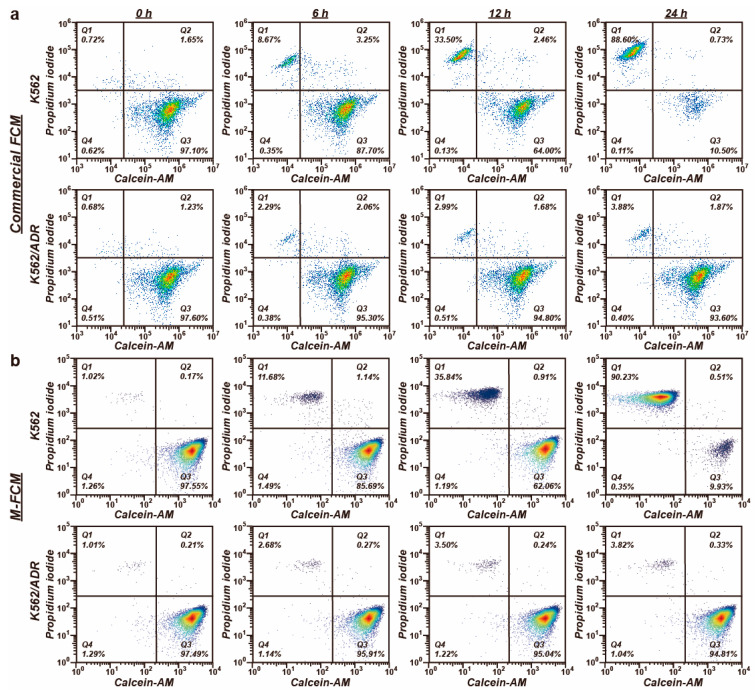
The scatter plots obtained by (**a**) the commercial FCM and (**b**) the M-FCM for K562 and K562/ADR cells over 24-h culture in the doxorubicin. Lower right: Positive Calcein-AM expression and negative PI expression (live cells). Upper left: Negative Calcein-AM expression and positive PI expression (dead cells).

The cell population was measured by both M-FCM and commercial FCM. Figure 6 shows the density scatter plots describing the two events for the cell status in the PI and Calcein-AM fluorescence channels. The plots are classified into four quadrants. The scattering points in the right lower quadrants are identified as cells with positive expression of Calcein-AM and low expression of PI, indicating that these cells were insensitive to the drug, while those in the left upper quadrant are identified as dead cells, which suffered from the drug stimulation. Regarding the K562 cell line, the population of the dead cells increased with the duration of the exposure to the drugs. After 24-h treatment, one cell in ten was still alive. The populations of the dead cells were 88.6% and 90.2%, as measured by the commercial FCM and the M-FCM, respectively. The variation in the K562/ADR cells, by contrast, was insignificant. Most of the cells remained alive after the one-day drug treatment. The populations of the live cells calculated by the commercial FCM and the M-FCM were 93.6% and 94.8%, respectively. This demonstrates that the K562/ADR cells were resistant to the doxorubicin. The M-FCM produced the same result as the commercial FCM. The Bland–Altman plot for the classification of cell status measured by the M-FCM and the commercial FCM is shown in Figure S9. In total, 31 out of 32 cases were in the 95% confidence interval, indicating that the classification and the population calculation obtained by the M-FCM agreed well with those provided by the commercial FCM.

## 4. Conclusions

In this study, we demonstrated a compact, reconfigurable FCM. The design of the M-FCM follows a standard for modularization. Each module is developed independently for a specific function. The detection modules can be flexibly updated to satisfy various applications of fluorescence detection. The in situ QD-based light source generates narrow-bandwidth light to excite samples. The emissions in the different fluorescence channels can be read out severally, avoiding the optical loss resulting from in-series optical filtering, as in the layout in the conventional FCM. Thanks to the optical isolation of the microchannels, optical crosstalk among the fluorescence channels is eliminated. The palm-sized M-FCM exhibits a high signal-to-noise ratio. In the statistics for the cell population for the drug screening, the M-FCM with three fluorescence channels was applied to count the cells with different statuses and evaluate the drug resistance of the cells. The measurement was repeatedly conducted for the drug treatment over different durations. The results were consistent with those obtained with the commercial FCM, demonstrating the accuracy and reliability of the M-FCM.

The proposed M-FCM is a customizable platform for the multivariate analysis of cellular characteristics. In addition to fluorescence detection, various modules for cellular characterization, e.g., electrical impedance measurement and mechanical phenotyping, can be jointly used to upgrade the M-FCM to study cells comprehensively. As a prototype of multivariate cytometry, the M-FCM has the potential to be employed in biomedical research and disease diagnosis.

## Data Availability

The data presented in this study are available on request from the corresponding author.

## Supplementary Materials

The following supporting information can be downloaded at: https://www.mdpi.com/article/10.3390/bios14080395/s1. Figure S1: Simulation of the 3D hydrodynamic focusing; Figure S2: Structure of the optical waveguide; Figure S3: Spectra of the UV LED and QD films; Figure S4: The optical spectra of the UV LED and the emission of the blue and green QD films; Figure S5: The spectrum of the resin used for the frame; Figure S6: The background noise measured in the single capillary tube when one of the light sources was turned on; Figure S7: The background noise measured in the M-FCM for different combinations of the detection modules when one of the light sources was turned on; Figure S8: The waveforms at exponential scale for the three fluorescence channels detected by the M-FCM for (a) K562 cells and (b) K562/ADR cells after 24-h culture in the doxorubicin.; Figure S9: The Bland–Altman plot for the classification of cell viability/death obtained by the commercial FCM and the M-FCM; Table S1: The optical crosstalk in the detection system among the fluorescence channels using a single capillary tube; Table S2: The optical crosstalk of the detection system among the fluorescence channels in the M-FCM with the combination of modules with excitation/emission values of 368/460 (Stage 1), 484/515 (Stage 2) and 525/620 (Stage 3); Table S3: The optical crosstalk of the detection system among the fluorescence channels in the M-FCM with the combination of modules with excitation/emission values of 368/460 (Stage 1), 525/620 (Stage 2) and 484/515 (Stage 3); Table S4: The optical crosstalk in the detection system among the fluorescence channels in the M-FCM with the combination of modules with excitation/emission values of 484/515 (Stage 1), 368/460 (Stage 2) and 525/620 (Stage 3); Video S1: The installation of the capillary tube in the 3D-printed detection module.
