# Supplementary material for: A Miniature Modular Fluorescence Flow Cytometry System
Source: Biosensors (Basel). 2024 Aug 16;14(8):395. doi: 10.3390/bios14080395 (PMC11353081; doi:10.3390/bios14080395)
Supplement: Supplementary file 1 [file biosensors-14-00395-s001.zip › Biosensors_Supplementary Material_Modular cytometry_Revised.docx]

Supplementary Materials

A miniature modular fluorescence flow cytometry

Shaoqi Huang ^1^, Jiale Li ^1^, Li Wei ^1^, Lulu Zheng ^1^, Zheng Shi ^2^, Shiwei Guo ^3^, Bo Dai ^1,^*, Dawei Zhang ^1,^* and Songlin Zhuang ^1^

^1^ Engineering Research Center of Optical Instrument and System, the Ministry of Education, Shanghai Key Laboratory of Modern Optical System, University of Shanghai for Science and Technology, Shanghai, 200093, China; shaoqi_huang@163.com (S.H.); lijiale1019@163.com (J.L.); weili911126@163.com (L.W.); llzheng@usst.edu.cn (L.Z.); slzhuang@usst.edu.cn (S.Z.)

^2^ Department of Gastrointestinal Surgery, The First Affiliated Hospital of Naval Medical University, Shanghai 200433, China; mdshizheng@outlook.com

^3^ Department of Hepatobiliary Pancreatic Surgery, The First Affiliated Hospital of Naval Medical University, Shanghai 200433, China; gestwa@163.com

***** Correspondence: daibo@usst.edu.cn (B.D.); dwzhang@usst.edu.cn (D.Z.)

1. Supplementary Videos

**Video S1** The installation of the capillary tube in the 3D printed detection module.

2. Supplementary Figures

**
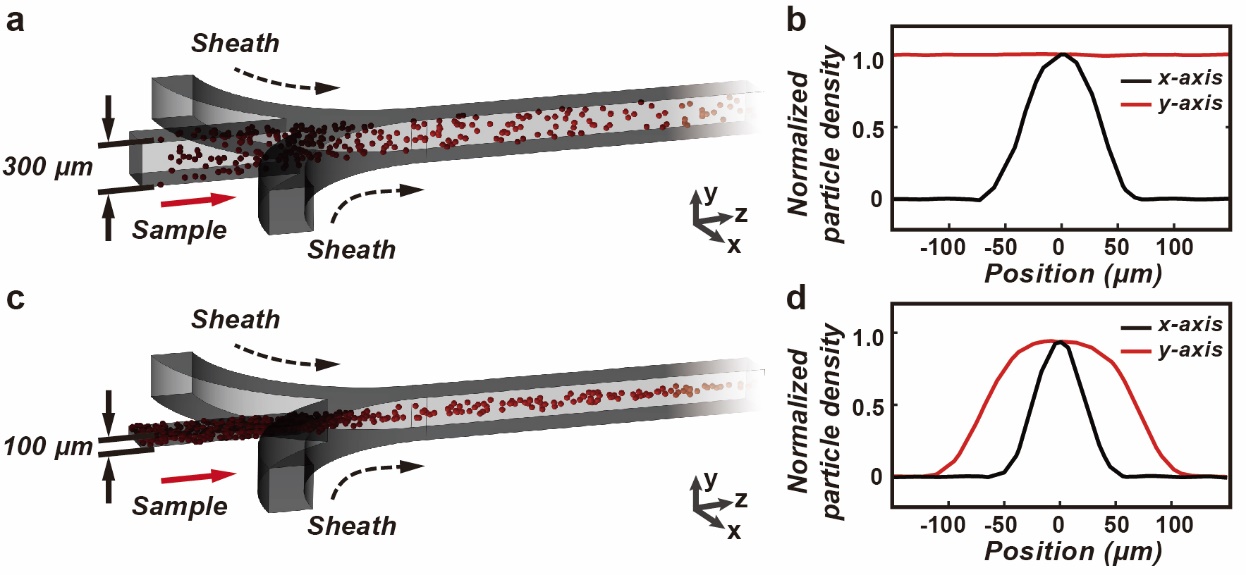
**

**Figure S1.** Simulation of the 3D hydrodynamic focusing. (**a**) and (**b**) Distribution of the particles when the injection channel for the particles has the height of 300 μm. The particles are hydrodynamically focused along x-axis but dispersed along z-axis; (**c**) and (**d**) Distribution of the particles when the injection channel for the particles has the height of 100 μm. The particles are focused along both x-axis and y-axis.

**
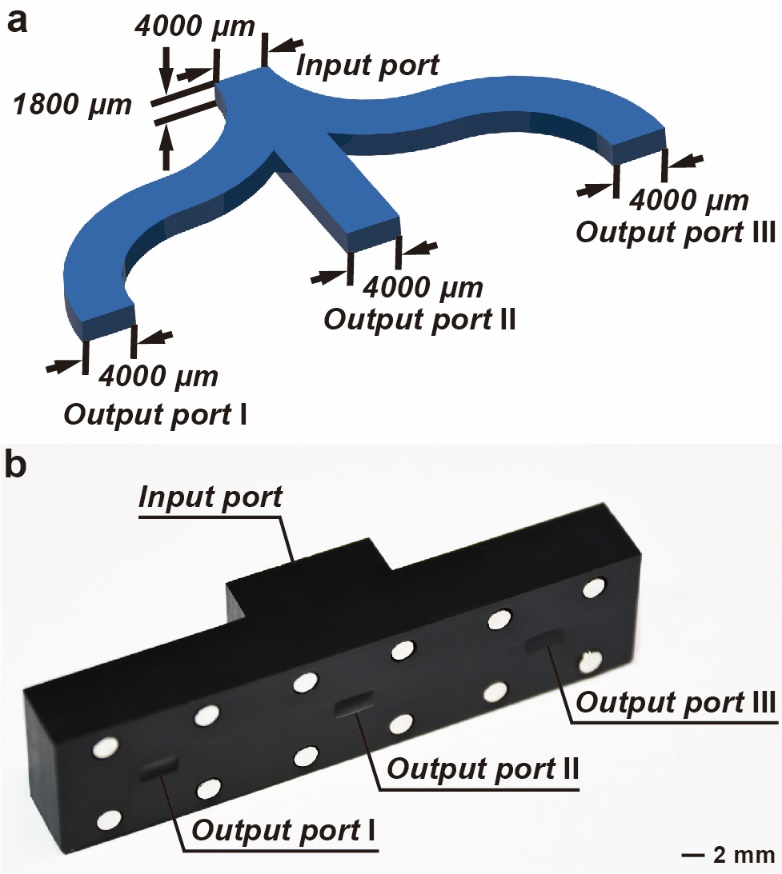
**

**Figure S2.** Structure of the optical waveguide. (**a**) Design of the 1×3 optical splitter; (**b**) Photograph of the illumination module.


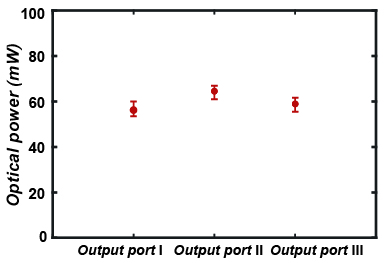


**Figure S3.** The optical power at the output ports of the optical splitter.


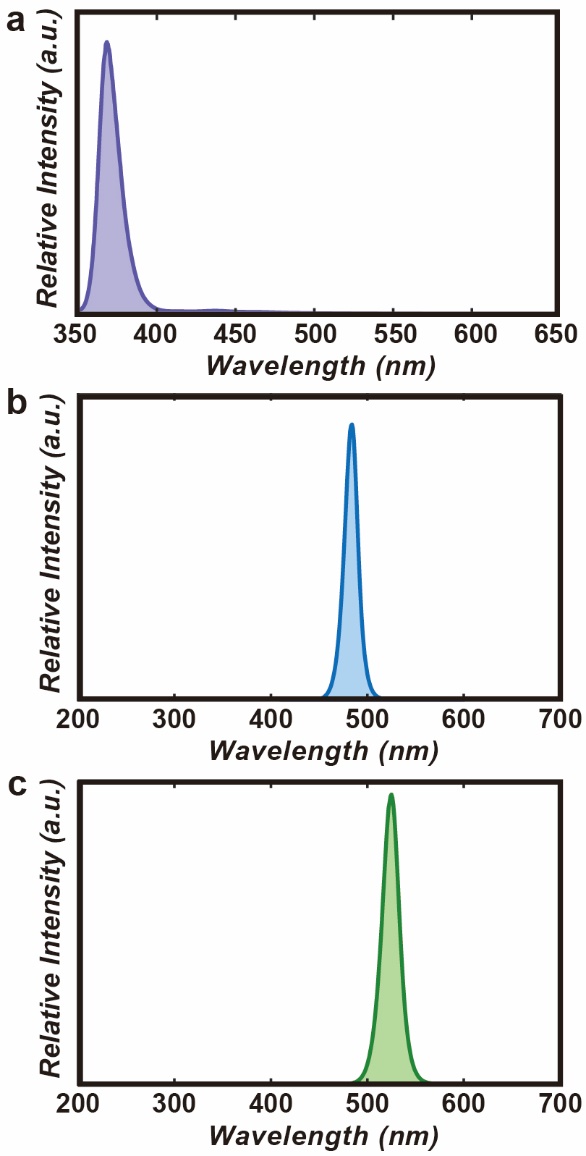


**Figure S4.** The optical spectra of the (a) UV LED, (b) the emission of the blue QD film and (c) the emission of the green QD film. The peak wavelength of the UV LED is 368 nm with the full-width at half-maximum bandwidth of 15 nm. The emission peaks of the blue QD film and the green QD film are at 484 nm and 525 nm with the full-width at half-maximum bandwidths of 16 nm and 19 nm, respectively.


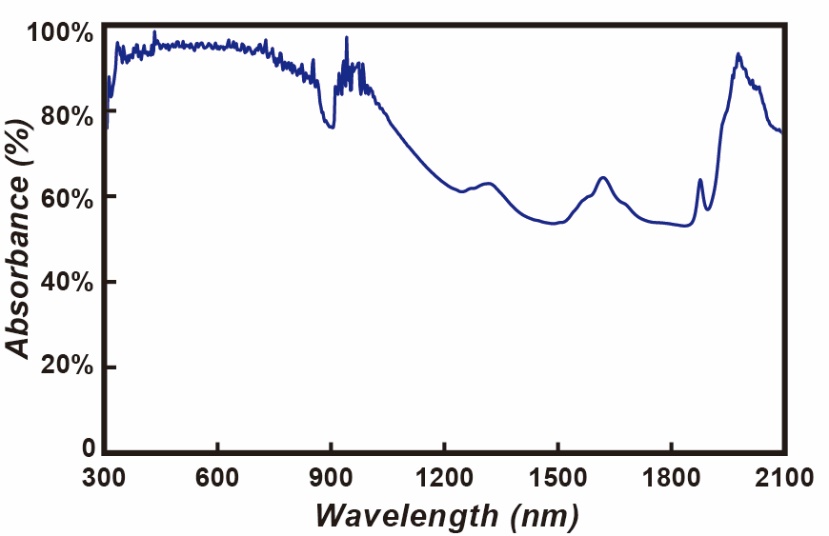


**Figure S5.** The spectrum of the resin used for the frame. The black resin has high absorbance (>90%) over the UV to visible band (365 nm to 800 nm).

**
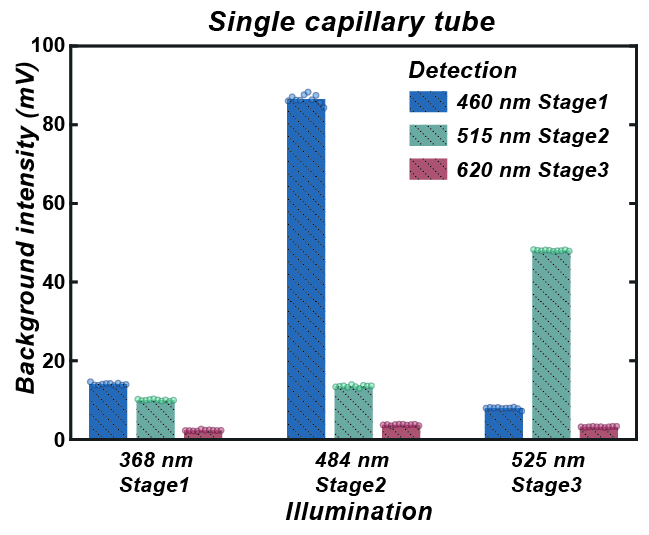
**

**Figure S6.** The background noise measured in the single capillary tube when one of the light sources was turned on.


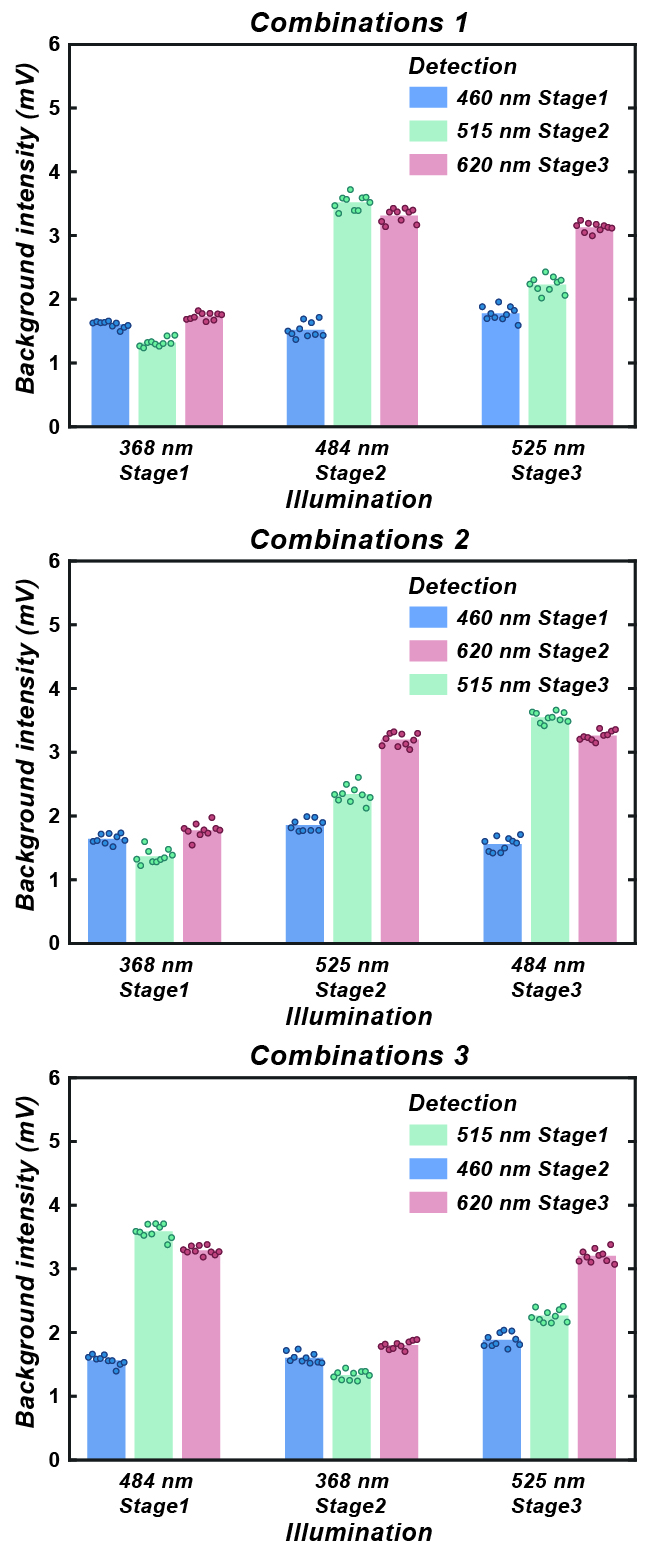


**Figure S7.** The background noise measured in the M-FCM for different combinations of the detection modules when one of the light sources was turned on. Combination 1: 368/460 (Stage 1), 484/515 (Stage 2) and 525/620 (Stage 3). Combination 2: 368/460 (Stage 1), 525/620 (Stage 2) and 484/515 (Stage 3). Combination 3: 484/515 (Stage 1), 368/460 (Stage 2) and 525/620 (Stage 3).


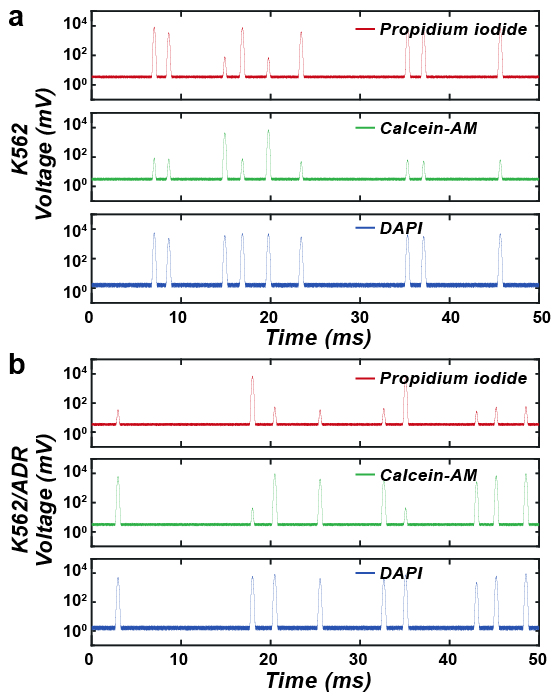


**Figure S8.** The waveforms in exponential scale for the three fluorescence channels detected by the M-FCM for (a) K562 cells and (b) K562/ADR cells after 24-hour culture in the doxorubicin.


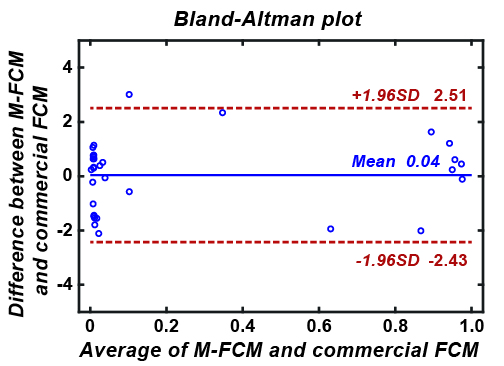


**Figure S9.** The Bland-Altman plot for the classification of cell viability/death obtained by the commercial FCM and the M-FCM. The blue solid line is the average of the difference, while the red dashed lines are the boundaries of the 95% confidence interval.

3. Supplementary Table

**Table S1.** The optical crosstalk of the detection system among the fluorescence channels using a single capillary tube.

| **Replication** |  |  | **Illumination** | | |
| --- | --- | --- | --- | --- | --- |
|  |  |  | Stage1 | Stage2 | Stage3 |
| Case 1 | **Detection** | Stage1 | 14.659 | 86.043 | 7.950 |
|  |  | Stage2 | 10.218 | 13.362 | 48.264 |
|  |  | Stage3 | 2.269 | 3.691 | 3.188 |
| Case 2 |  | Stage1 | 13.842 | 87.068 | 8.162 |
|  |  | Stage2 | 9.854 | 13.494 | 48.077 |
|  |  | Stage3 | 2.236 | 3.779 | 3.054 |
| Case 3 |  | Stage1 | 13.761 | 86.257 | 8.006 |
|  |  | Stage2 | 9.964 | 13.641 | 47.954 |
|  |  | Stage3 | 2.160 | 3.451 | 3.209 |
| Case 4 |  | Stage1 | 14.035 | 86.215 | 8.138 |
|  |  | Stage2 | 10.138 | 13.249 | 48.160 |
|  |  | Stage3 | 2.119 | 3.794 | 3.317 |
| Case 5 |  | Stage1 | 14.208 | 87.605 | 7.854 |
|  |  | Stage2 | 10.305 | 13.954 | 48.051 |
|  |  | Stage3 | 2.628 | 3.881 | 3.185 |
| Case 6 |  | Stage1 | 14.229 | 88.289 | 7.951 |
|  |  | Stage2 | 10.034 | 13.415 | 47.814 |
|  |  | Stage3 | 2.345 | 3.862 | 3.229 |
| Case 7 |  | Stage1 | 13.694 | 86.330 | 7.990 |
|  |  | Stage2 | 9.766 | 12.986 | 47.951 |
|  |  | Stage3 | 2.414 | 3.647 | 3.025 |
| Case 8 |  | Stage1 | 14.301 | 87.455 | 8.210 |
|  |  | Stage2 | 10.035 | 13.750 | 47.963 |
|  |  | Stage3 | 2.348 | 3.758 | 3.192 |
| Case 9 |  | Stage1 | 13.812 | 85.624 | 7.859 |
|  |  | Stage2 | 9.648 | 13.556 | 48.207 |
|  |  | Stage3 | 2.264 | 3.865 | 3.337 |
| Case 10 |  | Stage1 | 13.967 | 84.336 | 8.223 |
|  |  | Stage2 | 9.971 | 13.643 | 47.861 |
|  |  | Stage3 | 2.305 | 3.502 | 3.320 |

**Table S2.** The optical crosstalk of the detection system among the fluorescence channels in the M-FCM with the combination of modules with the excitation/emission of 368/460 (Stage 1), 484/515 (Stage 2) and 525/620 (Stage 3).

| **Replication** |  |  | **Illumination** | | |
| --- | --- | --- | --- | --- | --- |
|  |  |  | Stage1 | Stage2 | Stage3 |
| Case 1 | **Detection** | Stage1 | 1.628 | 1.501 | 1.882 |
|  |  | Stage2 | 1.267 | 3.469 | 2.239 |
|  |  | Stage3 | 1.686 | 3.221 | 3.157 |
| Case 2 |  | Stage1 | 1.649 | 1.460 | 1.697 |
|  |  | Stage2 | 1.237 | 3.347 | 2.305 |
|  |  | Stage3 | 1.698 | 3.139 | 3.240 |
| Case 3 |  | Stage1 | 1.632 | 1.369 | 1.776 |
|  |  | Stage2 | 1.322 | 3.589 | 2.169 |
|  |  | Stage3 | 1.719 | 3.368 | 3.047 |
| Case 4 |  | Stage1 | 1.638 | 1.537 | 1.714 |
|  |  | Stage2 | 1.334 | 3.568 | 2.020 |
|  |  | Stage3 | 1.820 | 3.428 | 3.193 |
| Case 5 |  | Stage1 | 1.659 | 1.691 | 1.958 |
|  |  | Stage2 | 1.297 | 3.721 | 2.430 |
|  |  | Stage3 | 1.776 | 3.372 | 2.997 |
| Case 6 |  | Stage1 | 1.580 | 1.427 | 1.694 |
|  |  | Stage2 | 1.264 | 3.394 | 2.156 |
|  |  | Stage3 | 1.649 | 3.242 | 3.176 |
| Case 7 |  | Stage1 | 1.627 | 1.633 | 1.759 |
|  |  | Stage2 | 1.306 | 3.392 | 2.351 |
|  |  | Stage3 | 1.779 | 3.429 | 3.091 |
| Case 8 |  | Stage1 | 1.495 | 1.448 | 1.883 |
|  |  | Stage2 | 1.426 | 3.591 | 2.268 |
|  |  | Stage3 | 1.673 | 3.367 | 3.154 |
| Case 9 |  | Stage1 | 1.559 | 1.716 | 1.825 |
|  |  | Stage2 | 1.308 | 3.601 | 2.299 |
|  |  | Stage3 | 1.770 | 3.399 | 3.130 |
| Case 10 |  | Stage1 | 1.589 | 1.434 | 1.592 |
|  |  | Stage2 | 1.436 | 3.520 | 2.062 |
|  |  | Stage3 | 1.758 | 3.167 | 3.117 |

**Table S3.** The optical crosstalk of the detection system among the fluorescence channels in the M-FCM with the combination of modules with the excitation/emission of 368/460 (Stage 1), 525/620 (Stage 2) and 484/515 (Stage 3).

| **Replication** |  |  | **Illumination** | | |
| --- | --- | --- | --- | --- | --- |
|  |  |  | Stage1 | Stage2 | Stage3 |
| Case 1 | **Detection** | Stage1 | 1.625 | 1.886 | 1.584 |
|  |  | Stage2 | 1.308 | 2.312 | 3.591 |
|  |  | Stage3 | 1.785 | 3.068 | 3.168 |
| Case 2 |  | Stage1 | 1.584 | 1.796 | 1.428 |
|  |  | Stage2 | 1.209 | 2.226 | 3.571 |
|  |  | Stage3 | 1.742 | 3.179 | 3.208 |
| Case 3 |  | Stage1 | 1.597 | 1.885 | 1.404 |
|  |  | Stage2 | 1.580 | 2.325 | 3.419 |
|  |  | Stage3 | 1.628 | 3.261 | 3.195 |
| Case 4 |  | Stage1 | 1.698 | 1.742 | 1.671 |
|  |  | Stage2 | 1.429 | 2.469 | 3.378 |
|  |  | Stage3 | 1.854 | 3.284 | 3.161 |
| Case 5 |  | Stage1 | 1.557 | 1.751 | 1.408 |
|  |  | Stage2 | 1.269 | 2.203 | 3.500 |
|  |  | Stage3 | 1.688 | 3.054 | 3.112 |
| Case 6 |  | Stage1 | 1.707 | 1.968 | 1.481 |
|  |  | Stage2 | 1.266 | 2.384 | 3.510 |
|  |  | Stage3 | 1.762 | 3.249 | 3.337 |
| Case 7 |  | Stage1 | 1.502 | 1.754 | 1.627 |
|  |  | Stage2 | 1.303 | 2.578 | 3.621 |
|  |  | Stage3 | 1.712 | 3.095 | 3.229 |
| Case 8 |  | Stage1 | 1.656 | 1.958 | 1.588 |
|  |  | Stage2 | 1.330 | 2.307 | 3.469 |
|  |  | Stage3 | 1.954 | 3.009 | 3.238 |
| Case 9 |  | Stage1 | 1.715 | 1.756 | 1.559 |
|  |  | Stage2 | 1.462 | 2.100 | 3.581 |
|  |  | Stage3 | 1.785 | 3.155 | 3.294 |
| Case 10 |  | Stage1 | 1.600 | 1.877 | 1.690 |
|  |  | Stage2 | 1.371 | 2.265 | 3.447 |
|  |  | Stage3 | 1.758 | 3.260 | 3.319 |

**Table S4.** The optical crosstalk of the detection system among the fluorescence channels in the M-FCM with the combination of modules with the excitation/emission of 484/515 (Stage 1), 368/460 (Stage 2) and 525/620 (Stage 3).

| **Replication** |  |  | **Illumination** | | |
| --- | --- | --- | --- | --- | --- |
|  |  |  | Stage1 | Stage2 | Stage3 |
| Case 1 | **Detection** | Stage1 | 1.580 | 1.684 | 1.759 |
|  |  | Stage2 | 3.511 | 1.279 | 2.190 |
|  |  | Stage3 | 3.229 | 1.746 | 3.054 |
| Case 2 |  | Stage1 | 1.628 | 1.528 | 1.885 |
|  |  | Stage2 | 3.499 | 1.343 | 2.351 |
|  |  | Stage3 | 3.194 | 1.781 | 3.195 |
| Case 3 |  | Stage1 | 1.552 | 1.580 | 1.758 |
|  |  | Stage2 | 3.450 | 1.223 | 2.160 |
|  |  | Stage3 | 3.287 | 1.696 | 3.115 |
| Case 4 |  | Stage1 | 1.561 | 1.706 | 1.789 |
|  |  | Stage2 | 3.621 | 1.415 | 2.109 |
|  |  | Stage3 | 3.207 | 1.715 | 3.039 |
| Case 5 |  | Stage1 | 1.618 | 1.522 | 1.957 |
|  |  | Stage2 | 3.471 | 1.226 | 2.264 |
|  |  | Stage3 | 3.295 | 1.791 | 3.251 |
| Case 6 |  | Stage1 | 1.527 | 1.572 | 1.995 |
|  |  | Stage2 | 3.628 | 1.336 | 2.106 |
|  |  | Stage3 | 3.117 | 1.752 | 3.143 |
| Case 7 |  | Stage1 | 1.528 | 1.490 | 1.705 |
|  |  | Stage2 | 3.574 | 1.216 | 2.210 |
|  |  | Stage3 | 3.309 | 1.669 | 3.164 |
| Case 8 |  | Stage1 | 1.368 | 1.626 | 1.983 |
|  |  | Stage2 | 3.627 | 1.361 | 2.309 |
|  |  | Stage3 | 3.195 | 1.815 | 3.061 |
| Case 9 |  | Stage1 | 1.472 | 1.505 | 1.857 |
|  |  | Stage2 | 3.306 | 1.364 | 2.361 |
|  |  | Stage3 | 3.149 | 1.841 | 3.308 |
| Case 10 |  | Stage1 | 1.502 | 1.495 | 1.774 |
|  |  | Stage2 | 3.415 | 1.302 | 2.120 |
|  |  | Stage3 | 3.199 | 1.851 | 3.006 |
